# A Multistate Trial of an Early Surveillance Program for Autism Within General Practices in Australia

**DOI:** 10.3389/fped.2021.640359

**Published:** 2021-04-23

**Authors:** Josephine Barbaro, Anne Masi, Melissa Gilbert, Radhika Nair, Ifrah Abdullahi, Joseph Descallar, Cheryl Dissanayake, John Eastwood, Iqbal Hasan, Bin Jalaludin, Lisa Karlov, Feroza Khan, Jane Kohlhoff, S. T. Liaw, Raghu Lingam, Antonio Mendoza Diaz, Natalie Ong, Chun Wah Michael Tam, Katy Unwin, Sue Woolfenden, Valsamma Eapen

**Affiliations:** ^1^Olga Tennison Autism Research Centre, School of Psychology and Public Health, College of Science, Health & Engineering, La Trobe University, Bundoora, VIC, Australia; ^2^Cooperative Research Centre for Living With Autism (Autism CRC), The University of Queensland, Indooroopilly, QLD, Australia; ^3^Faculty of Medicine, School of Psychiatry, University of New South Wales, Sydney, NSW, Australia; ^4^Ingham Institute for Applied Medical Research, Liverpool, NSW, Australia; ^5^South Western Sydney Clinical School, University of New South Wales, Sydney, NSW, Australia; ^6^Faculty of Medicine and Health, School of Medicine, University of Sydney, Sydney, NSW, Australia; ^7^Faculty of Medicine, School of Women and Children's Health, University of New South Wales, Sydney, NSW, Australia; ^8^Faculty of Medicine, School of Population Health, University of New South Wales, Sydney, NSW, Australia; ^9^Academic Unit of Psychiatry, Infant Child and Adolescent Mental Health Services, South Western Sydney Local Health District, Sydney, NSW, Australia; ^10^Population Child Health Research Group, Faculty of Medicine, School of Women's and Children's Health, University of New South Wales, Sydney, NSW, Australia; ^11^Children's Hospital Westmead Clinical School, University of Sydney, Sydney, NSW, Australia; ^12^Primary and Integrated Care Unit, South Western Sydney Local Health District, Liverpool, NSW, Australia

**Keywords:** autism (ASD), developmental screening, developmental surveillance, toddlers, primary care, general practitioner

## Abstract

**Background:** The early detection of developmental conditions such as autism is vital to ensure children can access appropriate and timely evidence-based supports, services, and interventions. Children who have undetected developmental conditions early in life are more likely to develop later health, developmental, learning, and behavioral issues, which in turn can have a cumulative effect over the life course.

**Methods:** The current protocol describes a multi-site, cluster randomized control trial comparing a developmental surveillance pathway for autism to usual care, using opportunistic visits to general practitioners (GPs). Units of randomization are GP clinics across two Australian states (New South Wales and Victoria), with thirty clinics within each state, each of which will aim to recruit approximately forty children aged between ~18- and 24-months, for a total of ~2,400 participants. Children will be randomized to two clusters; namely, an autism surveillance pathway (ASP) or surveillance as usual (SaU). The screening process for the ASP arm involves primary and secondary screenings for developmental concerns for autism, using both parent and GP reports and observations. Children in both arms who show signs of developmental concerns for autism will be offered a full developmental assessment by the research team at 24 months of age to determine the efficacy of developmental surveillance in successfully identifying children with autism.

**Trial Registration:** The trial is registered with ANZCTR (ACTRN12619001200178) and reporting of the trial results will be according to recommendations in the CONSORT Statement.

## Introduction

The increasing prevalence of developmental conditions such as autism, first evident in early childhood, can pose significant challenges to the individual, their family, and society if left undetected and unsupported (1, 2). It is estimated that over two hundred and fifty million children globally do not reach their developmental potential due to a lack of access to sufficient supports and services (3, 4). In Australia, around one in five children starting their first year of school are “developmentally vulnerable” with delays in one or more domains of development (5). Additionally, despite early signs of autism emerging within the first 2 years of life for most children (6), the average age of autism diagnosis in Australia is 4.1 years among children accessing intervention under the age of 7 years (7). Children who have undetected and unsupported developmental conditions early in life are more likely to develop later health issues, including adverse adult mental health outcomes (8, 9).

There is significant evidence that providing supports, services, and interventions early in the life course can enable children to reach their full potential (10). However, significant challenges and inequities remain in detecting children at high likelihood of developmental conditions such as autism sufficiently early to take advantage of neural plasticity of the developing brain through implementation of comprehensive, multimodal, evidence-based interventions (11). Specifically, early identification and optimal early intervention in the toddler, and preschool years for children with early signs of autism is critically important for optimizing outcomes. For example, it has been shown that only 8% of children who received a diagnosis of Autism Spectrum Disorder (ASD) at 2 years of age had comorbid Intellectual Disability (ID) when followed up at 9 years, while almost a quarter (24%) of those who received the diagnosis between 3- and 5- years were found to have co-morbid ID (12); this is highly significant, as the monetary cost of ASD *without* ID across an individual's lifespan is estimated to be US$1.4 million, while the cost of ASD *with* ID is almost doubled, at US $2.4 million (13). Further, children who begin school without developmental difficulties or delays will remain on educational trajectories that are enhanced in comparison to children who begin school with unaddressed developmental concerns (14–16).

Ongoing developmental surveillance offers opportunities to identify children with developmental differences reflecting early features of autism, in a systematic way through health and developmental promotion, and provide early intervention. Over the past three decades, data have emerged suggesting that developmental issues beginning in infancy and toddlerhood have the potential to affect key outcomes in the longitudinal trajectory (17, 18) and, the earlier intervention is commenced, the better the outcome (19). There is also increasing evidence to suggest that early detection and intervention is efficacious, cost-effective, and that it may be a way of decreasing health inequality, disparities, and breaking the cycles of intergenerational disadvantage (1, 20–22). However, it has been observed that, despite some parents having concerns about their child's development from a very early age, children who were diagnosed with ASD were identified later than children with developmental delay or intellectual disability (23). Such delay in diagnosis can also result in increased parental stress and significant delays in initiating early intervention which, as stated above, can result in less than optimal outcomes over time (24).

Whilst advances have been made in developing effective early interventions for ASD, significant barriers exist in accessing such intervention early within the period of greatest responsiveness to support and intervention (25). There are a few studies that have explored processes for the detection of developmental vulnerability in early life and barriers to accessing early intervention. One such study is the Watch Me Grow (WMG) program (26), conducted in a birth cohort of two thousand children in Sydney, Australia, in partnership with the New South Wales (NSW) state government health department. The WMG study (26–29) revealed that up to 30% of children are at developmental risk by their 18-month “well-child” check, but only 30–50% of these children attending primary health care (general practitioners—GPs, and child and family health nurses—CFHNs) have their developmental surveillance record completed. This is in keeping with the findings from a population health survey in NSW, Australia (30) that found that only around 66% of children attend “well-child” checks until around 12 months of age, and that this rate drops to 20% between 1 and 4 years of age. Thus, the period from 12 months to 5 years of age is a critical “silent” period for assessing a range of developmental and behavioral concerns, including speech and language difficulties and ASD. However, it is to be noted that across Australia there is considerable variability between states in children accessing developmental surveillance (26). For example, while NSW has a developmental surveillance attendance rate of 30% at 18 months (31), attendance is much higher in Victoria (VIC) at 74.2% (32).

Further, the WMG study found evidence of an “inverse care law,” whereby those at highest risk (such as children whose mothers were born overseas, or with lower maternal education and income levels) for developmental delay were the least likely to access the developmental surveillance program (27, 29). Qualitative analyses also indicated that facilitators to accessing developmental surveillance for families included proximity to the center, continuity of care, visits being combined with another recommended service (e.g., immunization) and language concordance by the provider (33). Access to good developmental surveillance programs could lead to better support for families, particularly those who have complex and multiple social risk factors.

The early detection of autism was investigated in Australia using Social Attention and Communication Surveillance (SACS) and its revised version (SACS-R) (34–37). A training program for universal developmental surveillance of autism was undertaken with maternal and child health nurses and early childhood educators, respectively. The program included education on the early behavioral markers for autism and the use of an evidence-based tool to monitor approximately thirty-four thousand children aged 8- to 24-months to identify those with a “high likelihood” of autism. Designed for use during routine developmental surveillance, the tool uses professional observations to monitor the child's development between 11- and 30-months of age using age-appropriate behavioral markers of autism (37). Children identified as having a “high likelihood” of autism were invited for developmental assessments by the research team, with six-monthly follow-ups and a “gold standard” diagnostic assessment at 24 months and 3.5 years of age. The SACS/SACS-R is currently the most accurate and sensitive tool for the early identification autism and related developmental conditions, such as developmental and/or language delay (psychometrics outlined in “Methods” section).

Currently there is considerable investment in disability services in Australia, with a new national program available for supporting those with a disability and their carers/families: the National Disability Insurance Scheme (NDIS) (38). The escalating costs, both financial and otherwise, of developmental conditions and particularly ASD has been highlighted by the fact that 29% of those entering the NDIS were doing so through a diagnosis of ASD (39). A significant proportion also have behavioral and emotional challenges, which, if not managed appropriately, can result in a lifetime of significant co-occurring mental health difficulties (33). This has led the Cooperative Research Centre for Living with Autism (Autism CRC) to provide funding support to carry out this study examining the implementation of a universal developmental surveillance program with a particular focus on ASD in the primary care setting. This study also aims to develop an integrated care pathway for follow up assessment, diagnostic determination, and referral to appropriate services, including secondary and tertiary services, in collaboration with the local health services as well as NDIS providers in the geographical area. This project will also help to evaluate the implementation of the recently launched National Health and Medical Research Council (NHMRC)-endorsed National Guideline for the Assessment and Diagnosis of Autism Spectrum Disorder in Australia (NAG) (40). Developed and published by the Autism CRC, with the financial support of the National Disability Insurance Agency (NDIA), the NAG aims to create greater consistency in diagnostic practices across Australia.

## Aims and Objectives

The overall objective of the project is to develop and evaluate a potentially sustainable approach to ASD detection within a developmental surveillance framework, in children aged 18–24 months using opportunistic GP clinic visits in the primary care setting. Specifically, this project will aim to examine whether, compared to Surveillance as Usual (SaU), an ASD Surveillance Pathway (ASP) will be associated with: (a) improved uptake and completion of developmental and autism surveillance; and (b) increased accuracy in identifying children at “high likelihood” of autism and related conditions, such as developmental and/or language delay. Secondary outcomes to be investigated include whether the ASD surveillance pathway will increase parental/caregiver engagement, health literacy, and satisfaction in accordance with the NAG, and increase GP awareness and utilization of developmental and autism surveillance tools and resources.

## Hypotheses

With regard to the primary outcomes, we hypothesize that, compared to SaU, the:

1. proportion of children completing autism developmental surveillance at the primary health care GP clinic will be increased in the ASP group
2. proportion of children correctly identified as being at “high likelihood” of autism, as evidenced by the comprehensive gold standard assessment results, will be increased in the ASP group.

Further, a qualitative component of the study will ascertain the parental/caregiver participation and experience in the program when followed up at 30-months-of-age. The study will also capture stakeholders' (including parents/caregivers and health professionals) perspectives of the barriers and enablers influencing the implementation of the NAG. The integrated model of developmental surveillance and referral used in the active arm of the intervention (ASP) will be compared to surveillance as usual (SaU) in terms of uptake of recommendations and service access and satisfaction, thereby informing the development of an integrated surveillance and care pathway in Australia. Thus, this study builds on existing state and national programs to identify barriers and develop mechanisms for accurate early detection of ASD in Australia.

## Methods and Analysis

### Study Design

**Figure 1:**
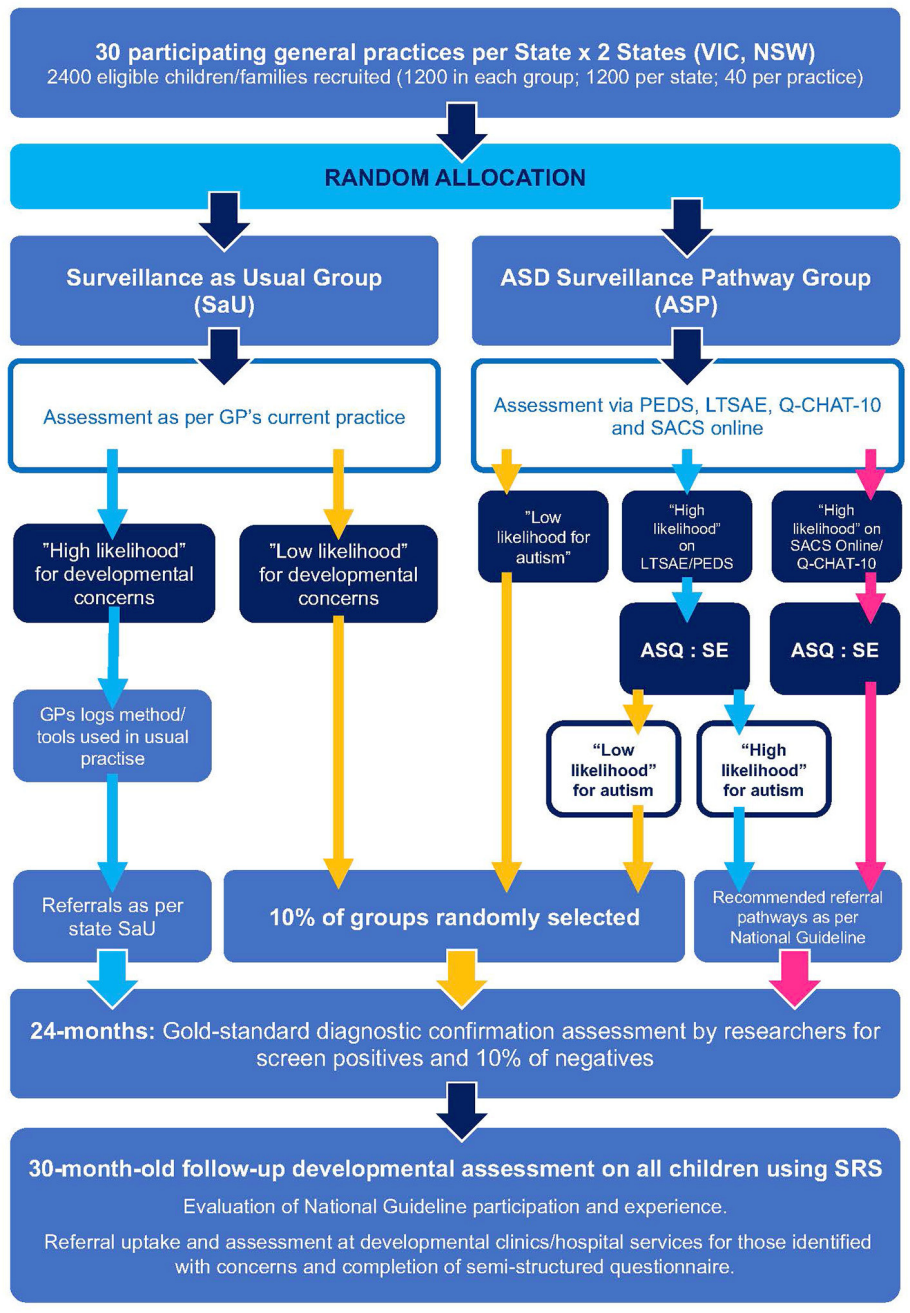
Study design.

A multi-site, cluster RCT with a qualitative component will be implemented (Figure 1). Using a minimization randomization procedure over size of GP clinic (<4 GPs or ≥4) and site (NSW or VIC), the participating GP clinics within the two Australian States (NSW and VIC) will be allocated to one of two arms:

1. Surveillance as Usual (SaU), representing current practice, or
2. ASD surveillance pathway (ASP), which will implement the “enhanced surveillance” protocol.

### Participants and Setting

Within each state (NSW, VIC), approximately thirty GP clinic “clusters” will be recruited (approximately sixty in total). Children aged approximately 18- to 24-months attending the GP clinics will then be recruited as part of each cluster. GP clinics will be recruited through various methods including using Primary Health Networks to distribute study information. GPs and, in some instances, the clinic practice nurse/s (PN) will be recruited, with each clinic having at least one GP consenting to recruit children to the study. A “universal surveillance” approach to recruitment will be implemented, such that any toddler between the eligible ages attending an appointment at the clinic for any reason, including for an immunization, can be recruited to the study. Approximately forty children will be recruited from each GP clinics, creating a total child sample size of ~2,400. For the qualitative component of the study, parents of children identified with a developmental concern and approximately twenty GPs and/or PNs will be recruited.

### Phase 1 - GP Clinic Visit

Prior to commencing the study, GPs and PNs will receive training on the study procedure and use of the study iPad and weblink, and, for those in the ASP group, the study screening tools. Clinic reception staff will also receive training on the study procedure and participant recruitment. Potential participants will be identified by reception staff when the child presents for their GP appointment, by staff checking the child's age in the patient record. Reception staff will offer the child's parent/caregiver information on the study in the form of a study flyer. Clinics will also have study posters with the same information on display. Parents/caregivers who would like to participate in the study will be asked to complete the participant information and consent form and demographic questions on the study iPad (or via their own mobile device), with those in the ASP group also completing developmental screening questionnaires. Once the parent/caregiver completes the developmental screening questionnaires and submits the response using the weblink, the GP receives the results via email or the Salesforce platform so that this information is available to the GP at the point of care during the consultation.

Table 1 summarizes clinical and behavioral measures and assessments for both ASP and SaU pathways. Measures will be taken at various points in the trial to allow appropriate comparisons between the groups (see Figure 1 and Table 1). Client-facing documents such as the information sheet, consent form, and screening questionnaires have been translated into Arabic, Chinese (Mandarin), and Vietnamese, the most common languages spoken by culturally and linguistically diverse (CALD) families in our study locations.

**Table 1:** Study Flow Chart.

**Time points**	**Age (months)**	**Location**		**Groups and tools**		
1	18–24		**Autism surveillance pathway**		**Surveillance as usual**	
			Service data		Service data	
		Waiting Room	Consent Demographics LTSAE; PEDS Q-CHAT-10		Consent Demographics	
		Doctor's Office	SACS Online (GP completes)		Brief online form to inform research team of high ASD risk, type of concern, screening method used, and referrals and recommendation provided	
			**Developmental concerns (any)**	**No concerns**		
		Waiting Room	ASQ:SE (option to complete at home)	–	–	
			**High ASD risk**	**Low ASD risk 10% complete**	**High ASD risk**	**Low ASD risk 10% complete**
2	24	Research Site	Demographics	Demographics	Demographics	Demographics
			ADOS-2	ADOS-2	ADOS-2	ADOS-2
			ADI-R	ADI-R	ADI-R	ADI-R
			MSEL	MSEL	MSEL	MSEL
			SEQ	SEQ	SEQ	SEQ
			ITSP	ITSP	ITSP	ITSP
			VABS-3	VABS-3	VABS-3	VABS-3
3	30	Online	SRS-2	SRS-2	SRS-2	SRS-2
			Semi-structured questionnaire		Semi-structured questionnaire	

*ADOS-2, Autism Diagnostic Observation Schedule Second Edition; ASD, Autism Spectrum Disorder; ADI-R, Autism Diagnostic Interview – Revised; ASQ:SE, Ages and Stages Questionnaire; ITSP, Infant Toddler Sensory Profile; LTSAE, Learn the Signs Act Early; MSEL, Mullen Scales of Early Learning; PEDS, Parents' Evaluation of Developmental Status; Q-CHAT, Quantitative Checklist for Autism in Toddlers; SACS-Online, Social Attention and Communication Surveillance – online; SEQ, Sensory Experiences Questionnaire - short form; SRS-2, Social Responsiveness Scale; VABS-3, Vineland Adaptive Behavior Scale 3rd Edition*.

### Measures

#### Autism Surveillance Pathway (ASP)

The parent completed developmental surveillance instruments are listed below.

“Learn The Signs Act Early” (LTSAE) developmental checks as recommended by the Centre for Disease Control (41) are used to monitor children's early development in the domains of social and emotional, language/communication, cognitive (learning, thinking, problem-solving), and movement/physical development. These developmental milestone checklists are available for children aged 2 months to 5 years, and the checklists for 18–23 and 24–29 months are included in this study. The aim of the LTSAE used by the NSW Health as part of the developmental surveillance program is to engage families and encourage parents/caregivers and health service providers to learn/monitor the signs of healthy development.

The Parents' Evaluation of Developmental Status (PEDS) (42) is designed for children from birth to 8 years of age and consists of ten questions asking parents/caregivers about concerns they have in domains including global/cognitive, expressive language and articulation, receptive language, fine and gross motor, behavior, self-help, socialization, and academic. It is widely used in Australia and is the first-line developmental surveillance tool used in state-based programs in most states in Australia and internationally. Screening test characteristics are over 70% for sensitivity and specificity for developmental concerns.

The Quantitative Checklist for Autism in Toddlers-10 item (Q-CHAT-10) is an ASD-specific ten-item tool to aid the decision making for primary care professionals about whether to refer a child for a full diagnostic assessment for ASD. The ten most accurate items from the original Quantitative Checklist for Autism in Toddlers (Q-CHAT) (43) were identified and included in this shortened version. Using a cut-off point of three, the Q-CHAT-10 has sensitivity of 0.91, specificity of 0.89, positive predictive value (PPV) of 0.58, and internal consistency of 0.85, when used in a *clinical population*; its utility when used in community-based samples has not been reported (44).

The Ages and Stages Questionnaire: Social Emotional scale (ASQ:SE) (45) is a screening tool focusing on the social and emotional behavior of children aged 6- to 60-months. This study uses the *18- and 24-month questionnaires*. Parents are asked a series of questions regarding their child's self-regulation, communication, autonomy, compliance, adaptive functioning, affect, and interaction with people. The ASQ (46) and the ASQ:SE have sound psychometric properties and the ASQ/ASQ-SE is the recommended developmental surveillance and health monitoring program in all Australian States with the exception of Victoria [where the Brigance Screens is used in its place (47)] for identification of developmental problems. The measure has been shown to have good reliability as a developmental screener globally with Cronbach's alpha of 0.79 for the total score and 0.61–0.74 for all the domains indicating acceptable reliability (48).

GP/PN completed autism developmental surveillance instrument:

The Social Attention and Communication Surveillance – Online (SACS Online) tool (34–37) is a professional observational tool intended for use in 11- to 30-month-old children, with the 18- and 24-month versions in use for this current study. Originally designed for use by maternal and child health nurses it is also suitable for use by other primary health professionals. The tool consists of five “key” behavioral items per age group plus additional items, with professionals asked to note whether the child is displaying typical or atypical behavior for each item. Where a child is noted to have atypical behavior for three of the five “key” items, it is recommended that they be referred for further assessment. Study results have indicated that the SACS tool and its revised version have high sensitivity (82–84%), specificity (99–99.8%), PPV for autism (81–83%; and 100% for *any* language/developmental delay or condition), and negative predictive value (99%), making it highly suitable for use in primary- and community-health and early childhood settings (34, 36). In the current study, GPs and PNs will be provided with access to the training modules in the use of the SACS Online tool via their online study portal, which they will complete prior to commencing child assessments.

#### Surveillance as Usual (SaU) Pathway

GPs and PNs will use a standard template to log the methods and tools used by the GP to assess children to be at high likelihood for autism, including details such as what concerns were raised and by whom, the assessment(s) completed, the reason for the assessment, the GP's findings, and any referrals and/or recommendations made.

### Procedures

#### Autism Surveillance Pathway (ASP)

Parents/caregivers in the ASP arm will complete electronic versions of a participant information and consent form, a brief demographic questionnaire, in addition to the LTSAE, PEDS, and Q-CHAT-10 in the GP clinic waiting room. REDCap (NSW site) and Salesforce (VIC) will be used to manage the completion of these forms and questionnaires. An embedded algorithm on the REDCap and Salesforce platforms will score the parent responses to identify children as having developmental risk and/or high likelihood for autism, and this information from the initial assessments will be passed on automatically to the child's GP (and PN if applicable). During the consultation, the GP (or PN) will also complete the SACS Online using the Salesforce platform at both NSW and VIC sites. The ASQ:SE will be completed by parents/caregivers of children identified as having any developmental concern following completion of the LTSAE, PEDS, Q-CHAT-10, and/or SACS Online. Children will be determined as having a “high likelihood” for ASD if they: (a) are identified as “high likelihood” for autism by either the Q-CHAT-10 *or* the SACS Online assessment (regardless of the outcome of the other tools); or (b) are identified as at developmental risk by LTSAE and/or PEDS *and* ASQ:SE (but were found not at high likelihood for autism by Q-CHAT-10 and SACS Online).

#### Surveillance as Usual (SaU) Pathway

Parents/caregivers of children in the SaU arm will complete electronic versions of the participant information and consent form and a brief demographic questionnaire. GPs (or PNs) will record any developmental screening method(s) used during the consultation using the standard template and the child's developmental risk status (i.e., high likelihood of autism).

### Phase 2 – 24-Month Assessment Protocol

#### Measures

##### ASD Traits

The Autism Diagnostic Observation Schedule-2 (ADOS-2) (49) will be used to confirm a diagnosis of ASD. The ADOS-2 is a semi-structured, standardized diagnostic observational assessment of social interaction, communication, play, and imaginative use of material for individuals suspected of having autism. The ADOS-2 module administered (by the trained clinician or researcher) is determined by the child's age and expressive language ability. The Toddler Module is for children aged between 12- and 30-months and will therefore be administered in this study (50, 51).

The Autism Diagnostic Interview-Revised (ADI-R) (52) is diagnostic, semi-structured, extended parental/caregiver interview including ninety-three items assessing reciprocal social interaction, communication, play, and repetitive, restricted, and sensory behaviors and interests. Test-retest and inter-rater agreement (intra-class correlations ≥ 0.92), and discriminant validity between autistic and non-autistic individuals for each of the domains are all excellent (52).

##### Developmental Skills

The Mullen Scales of Early Learning (MSEL) (53) is a standardized measure of nonverbal and verbal development in children from birth through to 68 months of age. The MSEL consist of five subscales: gross motor, fine motor, visual reception, receptive language, and expressive language. For each domain, raw scores and corresponding age equivalence scores are recorded. The gross motor scale will not be utilized in this study. The MSEL has excellent test-retest and inter-scorer reliability for children aged ≤ 24 months (*r* ≥ 0.82) (53).

##### Child Behavior

The Vineland Adaptive Behavior Scales third edition (VABS-3) (54), a parent/caregiver completed questionnaire, will be used to assess adaptive/functional skill development. It provides a measure of adaptive behavior in four broad domains of communication, daily living skills, socialization, and motor skills. The VABS-3 has sound psychometric properties with internal consistency of 0.90–0.98, test- re-test reliability of 0.80–0.92 and inter-rater reliability of 0.79 (55).

Parents and caregivers will also complete the Sensory Experiences Questionnaire - short form (SEQ) (56) and the Infant/Toddler Sensory Profile (ITSP) (57) to assess the child's sensory profile through understanding their response to a range of sensory experiences. The SEQ has good internal consistency (α = 0.80) (56) and excellent test-retest reliability (intra-class correlation = 0.92) (58). In seven to 36 month olds, the ITSP has poor to good internal consistency (α = 0.42–0.86) for item groupings, and acceptable to good test-retest reliability (intra-class correlation = 0.74–0.86) across sections and quadrants (59).

### Procedure

Parents/caregivers of children in both the ASP and SaU arms who are identified as having a “high likelihood” for autism at 18- or 24-months will be invited by the researchers for a developmental assessment when the child is ~24 months of age. Additionally, a randomly selected 10% of screen negatives from both groups will also be invited to complete this same assessment at ~24 months of age. These assessments will be undertaken by trained researchers who have achieved research-reliable coding on the ADOS-2 as well as having trained on the other measures described below. Parents/caregivers will complete a more detailed general and demographic questionnaire in addition to the following assessments: The ADOS-2 and ADI-R, together with the developmental assessment, and clinical judgement, will be used to determine whether the child meets a DSM-5 diagnosis of ASD.

### Phase 3 – 30-Month Assessment Protocol

Parents/caregivers of all children recruited to both the ASP and SaU arms of the study will complete the preschool version of the Social Responsiveness Scale, Second Edition (SRS-2)(60, 61) when their child is ~30 months of age. The SRS-2 is an autism screening tool that asks parents/caregivers to rate their child's traits of autism, as seen in a naturalistic setting, on a quantitative scale. The total score provides an indication of the extent of social differences. The SRS has good internal consistency (α = 0.93) and good test-retest reliability in preschoolers (intra-class correlation = 0.74) (61, 62).

Parents/caregivers of children identified with a developmental condition from both arms will also complete a semi-structured questionnaire to evaluate the uptake of recommendations, experience of assessment/service use, disability supports, and early intervention received and parental satisfaction with the health and disability services. This will facilitate comparison between those in the ASP arm who received the assessments and recommendations using the NAG and those in the SaU arm who received routine care.

### Phase 4 – Evaluation

GP clinic audits will be conducted to gather data on the number of children in the eligible age group seen during the recruitment period, the number of children where the developmental surveillance was conducted, and the number of children referred for developmental concerns, as per current practice. The data will be obtained using the clinics' client management software to generate deidentified reports on the numbers of children in the eligible age group seen during the recruitment period.

We will conduct qualitative interviews with 10 parent/caregiver and 10 professional (GPs/PNs) participants at each site in order to explore the enablers and barriers for completion of universal developmental and autism surveillance checks within the GP setting as well as about the utilization of the NAG, access to referral pathways and early interventions.

### Data Management

Data will be stored and managed at both sites using two applications – REDCap (Research Electronic Data CAPture) (63, 64) and Salesforce (65).

REDCap is a secure, web-based software platform designed to support data capture for research studies, providing (1) an intuitive interface for validated data capture; (2) audit trails for tracking data manipulation and export procedures; (3) automated export procedures for seamless data downloads to common statistical packages; and (4) procedures for data integration and interoperability with external sources. REDCap servers are located within Australia, thereby ensuring participant data is governed by Australian laws.

Salesforce is a highly secure cloud-based software application designed to store and manage customer data. The application is highly customizable and robust allowing a seamless experience to the end user through automation. The Salesforce database runs on force.com platform, which provides a powerful relational database. Salesforce is compliant with GDPR and Australian Data Privacy laws. It uses industry-accepted encryption products to protect customer data and communications during transmissions between La Trobe University network and the Covered Services, including through Transport Layer Encryption (TLS) leveraging at least 2048-bit RSA server certificates and 128-bit symmetric encryption keys.

Access to the data is restricted to members of the study team, with different levels of access for different members. If families request to complete questionnaires by hard copy, these will be sent to the family, with item level responses and summary and total scores entered in REDCap by the research team.

A unique identifier number for each of the participating children will be generated in REDCap/Salesforce when the parent/caregiver consents to participate in the study. Unique identifiers will also be generated when the SACS Online and ASQ:SE assessments are completed. This data will be assimilated onto the REDCap database and Salesforce database. Data generated at the NSW site will be managed using REDCap and data generated at VIC site will be managed using Salesforce.

### Analytical Plan

#### Sample Size

The total sample size of children recruited into the study will be 2,400 children across the two sites, which will provide adequate statistical power to address the study hypotheses. This sample will enable us to achieve 80% power to detect a difference between the two group means of 0.0935 standard deviations (SD) using a significance level of 0.05. In analyses comparing the smallest anticipated subgroups (as small as 20% of the original group sizes), a mean difference of 0.21 standard deviations will be detectable retaining the same power and significance levels. For the qualitative component of the study, it is envisaged that approximately twenty participants (GPs and PNs) will be enough to obtain thematic data saturation.

### Statistical Analysis

#### Quantitative Analysis

The entire raw dataset will be provided to an independent biostatistician, along with the study protocol and the pre-specified statistical analysis plan, who will conduct an independent analysis of the raw data. Details of this independent statistical analysis, as well as the name and academic institution of the independent statistician and whether compensation or funding was received for conducting the analyses, will be included in the publication of the results of the study.

Descriptive statistics for outcome measures (percentage, mean, and SD) and psychometric properties of the measures (reliability, Cronbach's α) will be reported. Descriptive statistics for all parental socio-demographic will also be presented, with the expectation of no group differences. We will compare the differences between the control arm (SaU) and the intervention arm (ASP) for the following primary outcomes:

(1a). the uptake of autism developmental surveillance as indicated by the proportion of children who participated in the program;
(1b). the proportion of children correctly identified by the GPs as being at “high likelihood” of autism, and later confirmed as having ASD through the “gold standard” assessment.
(2) the proportion of children in both groups who received referrals following identification of developmental concerns;
(3) the access and utilization of child health and developmental services will be ascertained based on the uptake of recommendations as assessed at the 30-month follow up.

analysis to determine the overall effectiveness of the “enhanced” autism surveillance pathway will involve comparison between the two groups, adjusting for potential confounders which are significantly different between clusters or strongly associated with the outcome variable, such as gender, maternal education, and socioeconomic status. Outcome group differences in the uptake of developmental surveillance, completion of surveillance tools and accurate identification of children at high likelihood of autism and diagnosed with ASD will be investigated using risk difference (RD) with 95% confidence interval (CI) and, where indicated, kappa coefficients. We will use generalized estimating equations (GEE) modeling to identify if there are factors other than the intervention that may also explain differences between the two arms. The intervention will be considered as a fixed effect and cluster assignment as a random effect, to account for group membership. Such modeling techniques adjust for clustering and also allow the inclusion of both cluster- and individual-level covariates. Statistical analysis will be carried out using IBM SPSS Statistics version 26 (66), Mplus 7.1 (67), and R v4.0.1 (68).

#### Qualitative Analysis

Interviews will be digitally audio recorded and transcribed verbatim, with transcripts analyzed using iterative thematic analysis (69) in qualitative software, NVivo (70). Identified themes will be compiled into a coding frame and, as new themes emerge, they will be compared against the initial coding frame, and either added as new themes, or used to expand and modify existing themes, until all data are accounted for. Data analysis will be undertaken using constant comparison methods and matrix displays will be used to explore similarities and differences across groups on key themes (71).

## Discussion

The early detection of autism is vital to ensure access to appropriate evidence-based supports, services, and early intervention during the critical period of neural plasticity to enhance later health, development and learning outcomes, and minimize behavioral issues. However, currently many children are missing early intervention opportunities. Further, our team has clearly demonstrated that there are inequities in access to early childhood development services which have led to the most disadvantaged populations, who have the highest likelihood of developmental concerns, being the least likely to engage with and access prevention and health promotion services such as developmental surveillance (29). Specifically, children from disadvantaged backgrounds have higher levels of unmet service needs but these families often struggle to understand and navigate the health system (72, 73). Further, families in rural settings face barriers of physical and social isolation, lack of availability of developmental services, limited financial resources and greater mental strain, each contributing to additional disadvantage for their children (74, 75). Hence, using the opportunistic contact families have with their GP to engage and empower them to monitor their children's developmental progress including any early signs of autism will no doubt improve early identification. There is an urgent need for a systematically driven program that is equitable, sustainable, and scalable across Australia, so that no child is left behind.

In addition, the current access, availability and quality of developmental services across Australia is further compounded by the fragmented and highly variable state-based health system (76). Given that all Australians have access to GP services, it is expected that by engaging GP clinics in the community and primary care settings, the current lack of access and engagement of parents/caregivers in developmental surveillance of their children will be successfully addressed. Further, by dove-tailing with the immunization schedule, which has high uptake, this program will be an example of “*proportionate universalism”*—the delivery of universal services coupled with targeted initiatives commensurate with need (77)—in action, regardless of socioeconomic, geographic, or cultural and linguistic backgrounds. The significance of the project is highlighted by the fact that there is increasing economic impact of ASD both from a healthcare and from a socio-economic perspective, in addition to the impacts on children and families. Early identification, support, and intervention can provide significant buffers to assist children on the autism spectrum to reach their optimum potential and enhance school readiness, as well as preventing a negative developmental trajectory and the entrenchment of difficulties. It is expected that this study will be of great benefit in the long term not only to children on the autism spectrum and their families, but also to the wider society, by decreasing the cascading economic impacts of later- and under-identification of autism, and the promotion of social participation, health, learning, and family outcomes.

## Ethics Statement

This study involved human participants. It was reviewed and approved by Human Research Ethics Committee University of New South Wales HC190143. Written informed consent to participate in this study was provided by the participants' parent or caregiver.

## Author Contributions

VE and JB initially designed the study protocol. AMa and MG contributed to subsequent amendments to the study protocol. AMa wrote the first draft of this manuscript. All authors contributed to subsequent drafts and approved the final manuscript.

## Conflict of Interest

The authors declare that the research was conducted in the absence of any commercial or financial relationships that could be construed as a potential conflict of interest.
